# Determining Malnutrition Assessment Criteria to Predict One-Year Mortality for Locally Advanced Head and Neck Cancer Patients Undergoing Concurrent Chemoradiotherapy

**DOI:** 10.3390/nu12030836

**Published:** 2020-03-20

**Authors:** Hang Huong Ling, Kun-Yun Yeh, Shu-Hang Ng, Cheng-Hsu Wang, Chien-Hong Lai, Tsung-Han Wu, Pei-Hung Chang, Wen-Chi Chou, Fang-Ping Chen, Yu-Ching Lin

**Affiliations:** 1 Division of Hemato-oncology, Department of Internal Medicine, Chang Gung Memorial Hospital, Keelung & Chang Gung University, College of Medicine, Keelung 204, Taiwanyehtyng@gmail.com (K.-Y.Y.); chw0098@gmail.com (C.-H.W.); lch2682@cgmh.org.tw (C.-H.L.); u402026@gmail.com (T.-H.W.); ph555chang@gmail.com (P.-H.C.); 2 Department of Medical Imaging and Intervention, Chang Gung Memorial Hospital, Linkou & Chang Gung University, Taoyuan 333, Taiwan; shuhangng@gmail.com; 3 Division of Hemato-oncology, Department of Internal Medicine, Chang Gung Memorial Hospital, Linkou & Chang Gung University, College of Medicine, Taoyuan 333, Taiwan; wenchi3992@yahoo.com.tw; 4 Department of Obstetrics and Gynecology, Chang Gung Memorial Hospital, Keelung 204, Taiwan; fangping@cgmh.org.tw; 5 College of Medicine, Healthy Aging Research Center, Chang Gung University, Taoyuan 333, Taiwan; 6 Osteoporosis Prevention and Treatment Center, Chang Gung Memorial Hospital, Keelung 204, Taiwan; 7 Department of Medical Imaging and Intervention, Chang Gung Memorial Hospital, Keelung & Chang Gung University, Keelung 204, Taiwan

**Keywords:** head and neck cancer, biomarkers, nutrition, survival, muscle mass

## Abstract

Study on the impact of pretreatment malnutrition on treatment outcomes in locally advanced head and neck cancer (LAHNC) patients is still lacking. We prospectively collected various malnutrition assessment methods including nutrition indexes, inflammatory biomarkers, and lean body mass index (LBMI) data before treatments. The one year mortality rate was assessed, and the factors associated with this outcome were investigated. Furthermore, the association between malnutrition assessment methods was examined. A total of 113 patients were enrolled. By prognostic stratification based on the prognostic nutritional index (PNI) and platelet-to-lymphocyte ratio (PLR) combination, the low PNI/high PLR group had highest and the high PNI/low PLR group had the lowest mortality rate. Furthermore, the PNI was positively correlated with the LBMI, and the PLR was inversely correlated with the LBMI. PNI and PLR were found to be independent prognostic factors of one year mortality and also associated with the loss of muscle.

## 1. Introduction

Malnutrition remains a major concern among patients with head and neck cancer (HNC), with a prevalence of 17–60% at the time of diagnosis [1,2,3,4]. Pretreatment malnutrition can manifest as a subacute or chronic condition that hampers the body composition and organ function, resulting in an increased risk of infection, deterioration of life quality, poor survival outcomes, and increased patient and care-related costs [2,5]. Thus, failure to obtain an early and rapid malnutrition diagnosis may result delayed nutritional support and consequently increased mortality in HNC patients.

Despite the various available assessment methods, standard diagnostic criteria for malnutrition are not well established in HNC patients [6]. The major obstacle to reach a diagnostic consensus is that different nutrition assessment criteria produce diverse malnutrition rates and statuses [7]. Moreover, different demographic characteristics, heterogeneous clinical staging, and disparate treatment modalities among studies may greatly influence the validation of the impact of malnutrition on HNC patient prognoses.

To establish reliable and practical diagnostic criteria for malnutrition, we conducted a prospective cohort observational study of patients with locally advanced head and neck cancer (LAHNC). The enrolled patients were stage III, IVA, or IVB patients who received standard concurrent chemoradiation (CCRT) at a single institution to minimize bias effects from a heterogeneous population and different treatment protocols. The reported 60 and 90-day short-term mortality for LAHNC patients after completion of CCRT ranged from 4.7% to 7.2% [8,9,10,11], but the one year mortality can escalate up to 25% [12,13,14]. Understanding the relevant nutritional assessment parameters for prediction of one year mortality in LAHNC patients will allow clinicians to identify malnutrition early and offer prompt action that may reduce mortality. Nevertheless, the literature concerning the influence of malnutrition on one year mortality in LAHNC is limited.

The aim of this study was to investigate the pretreatment nutritional status using various malnutrition assessment methods for prediction of one year mortality in LAHNC patients receiving CCRT and to examine the relationships between these nutritional parameters and the LBMI.

## 2. Materials and Methods

### 2.1. Study Design and Patients

An international panel of experts has suggested various methods to evaluate the nutritional status of HNC patients, including anthropometric, biochemical/hematological, and clinical/physical measurements [15,16,17]. Recently, non-invasive clinical imaging methods, such as dual energy X-ray absorptiometry (DXA), have been introduced to evaluate malnutrition, because they can precisely quantify the lean body mass with low radiation and costs [7,18,19]. This study evaluated commonly used clinical diagnostic parameters for malnutrition assessment, including: (1) nutrition indexes, such as the body mass index (BMI), body weight loss (BWL), prognostic nutritional index (PNI), nutritional risk index (NRI), and patient-generated subjective global assessment (PG-SGA); (2) inflammatory biomarkers, such as the neutrophil-to-lymphocyte ratio (NLR), platelet-to-lymphocyte ratio (PLR), and lymphocyte-to-monocyte ratio (LMR); and (3) the DXA-derived lean body mass index (LBMI).

Eligible patients were aged 20–75 years and had histologically proven locally advanced head and neck squamous cell carcinoma. Our patient populations had a variety of tumors originating from the oral cavity, oropharynx, hypopharynx, and larynx and metastatic cervical squamous cell carcinoma of unknown primary (CUP) site. The disease was staged according to the 7th edition of the American Joint Committee on Cancer Staging System (AJCC), which included stages III (T1-2, N1, or T3, N0-1), IVA (T4a, N0-1 or T1-4a, N2), and IVB (any T, N3 or T4b, any N). The clinical or pathological stages were reviewed and confirmed by multidisciplinary head and neck cancer specialists at our institute. The specialists included head neck surgeons, medical oncologists, radiation oncologists, nuclear medicine physicians, radiologists, and pathologists. Patients were included if they had an Eastern Cooperative Oncology Group (ECOG) performance status ≤ 2 with adequate hematopoietic or organ function and were amenable to concurrent chemoradiotherapy (CCRT). Patients were excluded if they had one of the following conditions: major gastrointestinal disorder, autoimmune disorder, end-stage renal failure, liver cirrhosis with intractable ascites, heart failure with New York Heart Association Classification IV, uncontrolled diabetes mellitus, ongoing infection, or receipt of regular medications that could substantially modulate metabolism or weight, such as steroids or megestrol acetate.

This prospective cohort study was carried out from February 2015 to November 2017. This study was approved by the institutional review board (IRB) of Chang Gung Memorial Hospital, Taiwan (IRB approval number: 103-3365A3) and was performed in accordance with good clinical practices and the Declaration of Helsinki guidelines. Written informed consent was provided by all patients. This trial was registered at ClinicalTrials.gov (identifier: NCT02854735).

### 2.2. CCRT Schedule

Curative CCRT alone was scheduled for patients with unresectable disease or organ preservation. Adjuvant CCRT were scheduled for patients after surgery if they had (1) 1 of 2 major risk factors (extranodal extension or a positive surgical margin) or (2) at least 3 of the following minor risk factors: pT4, pN1, close margin ≤ 4 mm, poor differentiation, perineural invasion, vessel invasion, lymph invasion, or depth ≥10 mm. CCRT was performed with radiotherapy delivered at a dose of 64–72 Gy in 32–36 fractions over an 8 week period, and concurrent chemotherapy with cisplatin (40 mg/m^2^) was administered once weekly. Neoadjuvant chemotherapy was allowed for patients before starting CCRT according to the treatment recommendations formulated by the head and neck cancer group in our institute. Neoadjuvant chemotherapy was performed with docetaxel (60–75 mg/m^2^) on Day 1, cisplatin (60–75 mg/m^2^) on Day 1, plus continuous infusion of 5 FU (1000 mg/m^2^/day) on Days 1–4 every 3 weeks.

All patients received routine hydration, antiemetic medications, and adequate supportive care. All patients were routinely referred to an early and intensive nutrition support program established in 2007 in our institute that included biweekly dietitian visits, mandatory feeding tube placement if the body weight loss was more than 5% during the treatment course, timely caloric supplementation, and blood transfusion as needed [20]. More than 90% of the patients remained in the hospital for completion of the treatment course and had government healthcare support via the Taiwan National Health Insurance Program.

### 2.3. Clinicopathological Data

Clinicopathological data were collected, including age, gender, body height, body weight, ECOG performance status, comorbid diseases, location, World Health Organization (WHO) histologic grade, AJCC 7th edition of tumor node metastasis (TNM) stage, treatment modality, including the chemotherapy and radiotherapy doses, and history of exposure to smoking, alcohol, and betel nuts. Participants were considered smokers if they currently smoked tobacco or had smoked in the past. Participants were considered alcohol drinkers if they reported consuming alcohol four or more times per week. Participants were considered betel quid users if they reported taking this substance during the previous year. The severity of comorbid diseases was scored using the head and neck Charlson Comorbidity Index (HN-CCI), which assessed the presence of heart failure, pulmonary disease, cerebrovascular disease, peptic ulcer, liver disease, and diabetes [21].

### 2.4. Malnutrition Assessment Methods

The nutrition indexes were evaluated using anthropometric methods, including the BMI and BWL, laboratory methods, including the PNI and NRI, and clinical assessment with the PG-SGA. BMI was defined as weight in kilograms divided by height in meters squared (kg/m^2^); BWL was calculated as (BW at diagnosis - ideal BW)/ideal BW × 100%. The laboratory nutrition indexes were calculated as follows: PNI, 10 × serum albumin (g/dL) + 0.005 × total lymphocyte count (TLC) (/mm^3^), and NRI, 1.519 × serum albumin (g/L) + 41.7 × (initial BW/ideal BW) [22,23]. The NRI scores were classified into severe (< 83.5), moderate (83.5–97.5), mild (97.5–100), and no malnutrition (> 100) risk groups [23]. The PG-SGA scores ranged between 0 and 35, with scores of 0–3 indicating well nourished, 4–8 indicating moderately malnourished, and ≥ 9 indicating severely malnourished [24].

The inflammatory biomarkers tested in this study included the NLR, PLR, and LMR. The NLR was defined as the ratio of the absolute neutrophil count to lymphocyte count, the PLR as the ratio of the platelet count to lymphocyte count, and the LMR as the ratio of the lymphocyte count to monocyte count. For lean mass measurement, DXA (GE Medical System, Lunar iDXA, Madison, WI, USA) was performed with the patients in the supine position on the table according to the manufacturer’s protocol. The scan modes (standard, thin, or thick) were automatically selected by the scanner software depending on the body size and BMI. Scans were analyzed using the enCORE Software, Version 15 (GE Lunar). The LBMI was calculated as lean mass in kilograms divided by height in meters squared (kg/m^2^).

All anthropometric measurements, blood tests, and DXA studies were completed 1 week prior to CCRT initiation. The 1 year mortality rate was defined as the proportion of patients who died within 365 days after CCRT initiation, which was used as the reference date due to variation from the time for stage workups.

### 2.5. Statistical Analysis

The statistical analyses were performed using SPSS Version 22.0 (SPSS Inc., Chicago, IL, USA). We assumed that the one year mortality rate was 25% with a power of 80% and α error of 0.05; the calculated sample size was at least 108. If the attrition rate was 10%, then the total number of patients that needed to be recruited was 120. Receiver operating characteristic (ROC) curves were used to determine the optimal cutoff values for the PNI, NLR, PLR, and LMR. The lowest quartile was used as the cutoff value for the LBMI [25,26], because no standard reference value was available. The associations between different clinicopathological and nutritional parameters and the one year mortality rate were analyzed by Cox proportional hazard models by applying predefined cut-off values. Forward stepwise selection was used in the univariate and multivariate analyses for different nutritional parameters. All independent variables significantly associated with the one year mortality rate (*p* < 0.05) in the univariate analysis were included in the multivariate analysis. Mortality differences among groups stratified by the PNI/PLR classification were further examined by logistic regression. The associations between nutrition indexes or inflammatory biomarkers and the LBMI were examined by Pearson’s or Spearman’s rank correlation coefficient after all variables were assessed using the Kolmogorov–Smirnov normality test. All differences were considered statistically significant with a *p*-value < 0.05 (two-tailed).

## 3. Results

### 3.1. Patients

**Figure 1:**
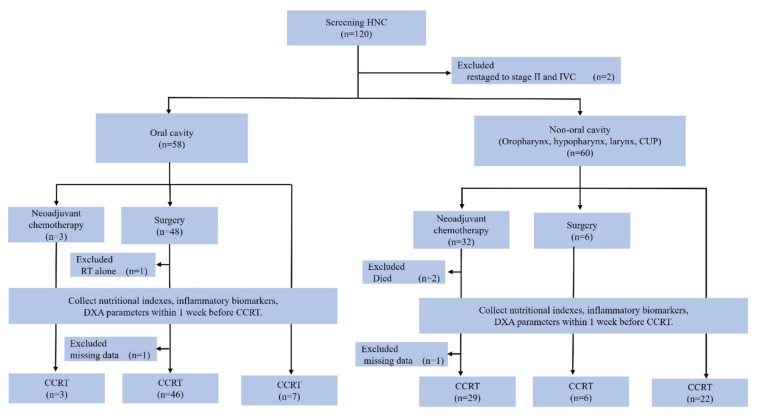
CONSORT diagram. Patients were considered to have completed planned therapy if they received at least four cycles of weekly cisplatin at 40 mg/m^2^ concomitants with radiotherapy (64–72 Gy). Incomplete data indicated patients who did not complete the DXA examination or missed blood tests. HNC, head and neck cancer; CUP, cancer of unknown primary; RT, radiotherapy; DXA, dual-energy X-ray absorptiometry; CCRT, concurrent chemoradiotherapy.

A total of 120 LAHNC patients were recruited, of whom 113 were eligible for analysis in this study. The patient enrollment, allocation, treatment modality, and data collection details are presented in a CONSORT diagram (Figure 1). The baseline patient characteristics are summarized in Table 1. Among these patients, the male gender was predominant, and the oral cavity (49.6%) was the most common tumor site. A high proportion of patients reported smoking (92.9%), alcohol (75.2%), and betel nut (62.8%) exposures. Among the 113 patients, 29 (25.7%) underwent CCRT alone, 52 (46.0%) received CCRT after curative surgery, and 32 (28.3%) underwent neoadjuvant chemotherapy followed by CCRT. At the one year follow-up after CCRT completion, 35 patients (30.9%) had died. The majority of the causes of death were related to cancer progression (71.4%), followed by severe infection (22.8%), and choking-related in-hospital cardiac arrest (3.6%).

### 3.2. Parameters Predicting One Year Mortality

Different malnutrition assessment methods produced different malnutrition rates, with a range from 15.0% based on a BMI < 18.5 kg/m^2^ to 39.8% based on a TLC < 1.5 × 10^9^/L (Supplementary Table S1). The univariate analysis revealed that the performance status, tumor location, curative surgery, hemoglobin, TLC, albumin level, BMI, PNI, NRI, NLR, PLR, LMR, and LBMI were significantly associated with the one year mortality rate of LAHNC patients (Table 2). After inclusion of these significant factors in the multivariate Cox proportional hazard model to adjust the effects of covariates, both low PNI (< 46.8; hazard ratio [HR] = 0.276; 95% CI, 0.131 to 0.582; *p* = 0.001) and high PLR (≥ 191; HR = 3.205; 95% CI, 1.520 to 6.757; *p* = 0.002) were identified as independent prognostic factors for prediction of the one year mortality of LAHNC patients (Table 2). Both PNI and PLR were also tested for multicollinearity in the logistic regression model, and both showed a low degree of correlation.

**Figure 2:**
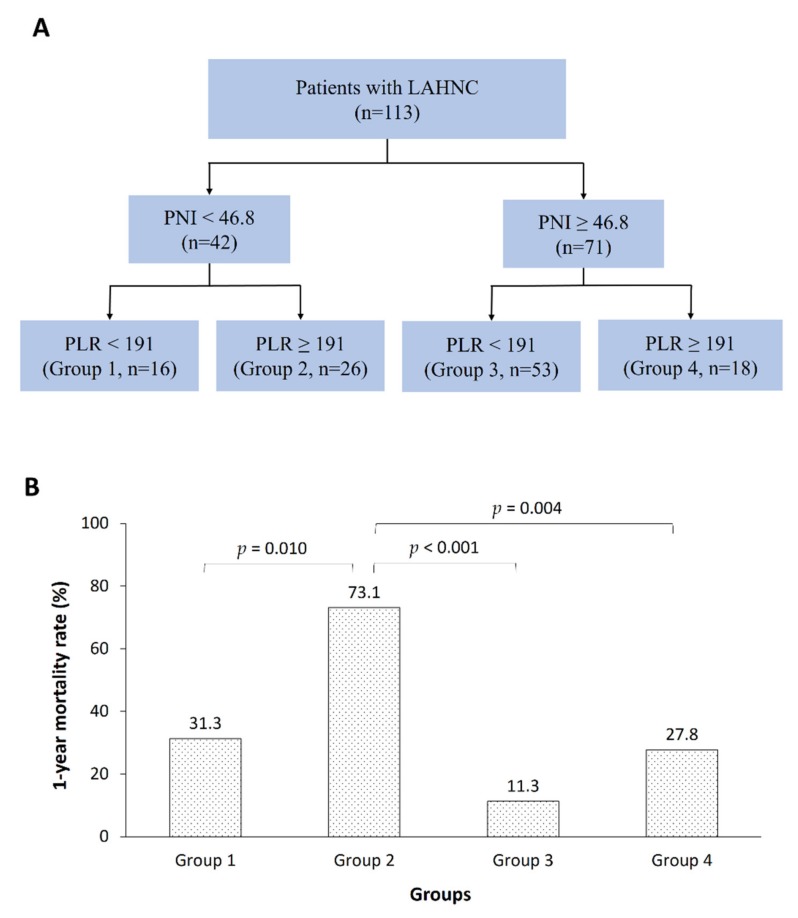
Risk groups based on the PNI and PLR combination predict the one year mortality rates of LAHNC patients undergoing CCRT. (**A**) Risk group stratification based on the PNI and PLR combination. (**B**) The one year mortality rates of the different risk groups by using the logistic regression model, adjusted for location, surgery, hemoglobin, TLC, albumin, BMI, NRI, and LBMI. LAHNC, locally advanced head and neck cancer; PNI, prognostic nutritional index; PLR, platelet-to-lymphocyte ratio; CCRT, concurrent chemoradiotherapy.

We further stratified the patients into four risk groups based on the PNI and PLR as follows: Group 1, low PNI/low PLR (*n* = 16); Group 2, low PNI/high PLR (*n* = 26); Group 3, high PNI/low PLR (*n* = 53); and Group 4, high PNI/high PLR (*n* = 18). Significant differences in the one year mortality rate were found among the groups by using logistic regression and the Kaplan–Meier survival curve, adjusted for location, surgery, hemoglobin, TLC, albumin, BMI, NRI, and LBMI. (Figure 2, Supplementary Figure S1). Patients in Groups 2 and 3 had the highest and lowest mortality rates (73.1% vs. 11.3%), respectively, and the difference between these two groups was significant (OR = 21.262, 95% CI, 6.317 to 71.562; *p* < 0.001). Moreover, a significantly higher mortality rate was observed for the patients in Group 2 than for those in Group 1 (OR = 0.167, 95% CI, 0.043 to 0.657; *p* = 0.010) and Group 4 (OR = 0.142, 95% CI, 0.037 to 0.545, *p* = 0.004). No difference in mortality was observed between Groups 1 (31.3%) and 4 (27.8%).

### 3.3. Relationships between the PNI or PLR and LBMI

Linear associations were found between the PNI or PLR and LBMI (Supplementary Figure S2). The PNI had a positive correlation with the LBMI (r = 0.198, *p* = 0.036), whereas the PLR had a negative correlation with the LBMI (r = −0.267, *p* = 0.004). When the patients were further stratified into survivor and non-survivor groups, the PNI still exhibited a positive correlation with the LBMI in the surviving patients (r = 0.224, *p* = 0.049), but not in the non-surviving patients. In contrast, the PLR was negatively associated with the LBMI in the non-surviving patients (r = −0.434, *p* = 0.009), but not in the surviving patients.

## 4. Discussion

Researchers have increasingly focused on establishing an easy and practical indicator to assist clinicians with early identification of treatment outcomes associated with the malnourished status. Our current study demonstrated two independent prognostic factors (PNI and PLR) that could be applied to LAHNC patients receiving CCRT. The PNI as the ratio of albumin and lymphocyte reflects both the nutritional and immunological conditions of a cancer patient [27,28]. Albumin is considered to stabilize cell growth and DNA replication, buffer biochemical changes, and maintain sex hormone homeostasis to protect against cancers [29]. Hypoalbuminemia is associated with chronic malnutrition and promotes the release of inflammatory cytokines [30]. Lymphocytes suppress tumor through inhabitation of dendritic cell activation and regulate T-cells and tumor associated macrophages [29]. Lymphopenia is associated with activation of cytotoxic immunity [31]. The PLR reflects the systemic inflammatory condition of a cancer patient. A high PLR value indicates thrombocytosis and/or lymphopenia. Thrombocytosis may dampen the recognition and cytotoxicity of tumor cells by immune cells [28] or promote tumor growth by stimulating tumor angiogenesis, vascular invasion, and distant metastasis [32,33]. Our results reinforce the predictive values of PNI and PLR seen in previous retrospective studies, which showed that the PNI predicted overall survival and treatment-related toxicities in LAHNC patients [22,34] and that the PLR predicted survival outcomes and disease recurrence in HNC patients [35,36,37]. Moreover, this study demonstrated that the combination of these two parameters provided better prediction of one year mortality in LAHNC patients. The mortality rate of patients with a low PNI and high PLR reached 73.1%; this finding consolidated the fact that poor nutrition and elevated inflammation may reduce the survival rate of LAHNC patients. It was evident that the patient’s nutritional, immunological, and inflammatory conditions determined the survival of the cancer patient. The combination of PNI and PLR biomarkers could provide an overview of all three conditions in the cancer patient and accurately predict the survival rate of cancer patients [38]. Moreover, peripheral blood cell counts (such as neutrophil, platelet, and monocyte count) alone are not well associated with survival, but by combining these cell counts into inflammatory biomarkers, these provide a more detail assessment of the inflammatory condition within the cancer patient [39].

Lean body mass is considered to be responsible for the distribution, metabolism, and clearance of antineoplastic agents. Patients with low LBMI are commonly intolerant to chemotoxicity. The increased treatment toxicity may be linked to systematic inflammation and, consequently, nosocomial infections and other complications and even death [40]. Thus, the investigation of the associations among the PNI, PLR, and LBMI may provide us a better understanding of how the nutritional, immunological, and inflammatory conditions affect the LBMI. Although the interplay between these three nutritional parameters in LAHNC patients undergoing CCRT remains unclear, the following evidence may shed light on their intricate interactions. First, the serum albumin level changes correspond to muscle mass alterations in healthy adults [41,42]. Muscle mass may serve as a dominant reservoir for albumin, and a low serum albumin level may enhance activation of oxidative damage in the muscle to release more albumin [41,42]; therefore, a positive correlation was found between the PNI and LBMI. Moreover, increased PLR values were significantly associated with sarcopenia and negatively correlated with the skeletal muscle index in geriatric and gastric cancer patients [43,44]. Since the PLR is an important indicator of the systematic inflammatory status and elevation of inflammatory cytokines will induce muscle wasting, enhance protein catabolism, and inhibit muscle synthesis [43,44], a negative correlation was found between the PNI and LBMI. In our study, the patients with high PNI values may have better muscle mass to compensate for the muscle breakdown induced by systemic inflammation from cancer or CCRT. Hence, these patients are more equipped to survive through treatment [45,46,47]. Conversely, patients with a low PNI have a lower muscle mass and may have difficulty overcoming the loss of muscle mass from inflammation and CCRT. Consequently, these patients are not able to hinder cancer progression or tolerate treatment and die as a result [45,46,47]. Moreover, the PNI was only positively correlated with the LBMI in the survivor group, but not in the non-survivor group. Therefore, we propose that the muscle mass was almost depleted in the non-survivor group patients, resulting in unstable provision of albumin from the muscle reservoir. This scenario may explain the loss of association between the muscle and PNI. Conversely, in the non-survivor group, ongoing systematic inflammation may further induce muscle wasting and break down; therefore, the PLR values were still negatively associated with the LBMI in this group.

Our work had some limitations. The comprehensiveness of the nutrition assessment remains a concern. C-reactive protein (CRP) was not included, although this measure might be elevated in malnourished patients and during HNC treatment [7]. Because CRP is interrelated with other nutritional or inflammatory parameters and the treatment modality, its prognostic effect on HNC patients is less commonly addressed [7]. Some anthropometric indexes (mid-arm circumference and triceps skinfold), muscle function tests (handgrip strength and physical activity), and body composition analyses using technology (bioelectrical impedance analysis and computed tomography scan) have been studied in HNC patients [7,48,49]. However, whether the addition of more nutritional or inflammatory parameters to the current model will provide better predictions is unclear. Additionally, administering cisplatin at 100 mg/m^2^ once every three weeks offered better locoregional tumor control and overall survival than administration at 30 mg/m^2^ once weekly [50]. Since the major cause of mortality in our study was cancer progression, selection of the drug regimen could also have contributed to the higher one year mortality rate in our study than in previous reports [12,14]. Therefore, we speculated that different chemotherapy regimens might also alter the significance of the nutritional/inflammatory parameter profiles for the treatment outcome. Lastly, this study’s results were based on a small sample size and short duration of follow-up time. Replication of study with larger population size and longer follow-up time is warranted before making a strong statement about the study results.

## 5. Conclusions

In conclusion, LAHNC patients with nutrition risks need to be identified as early as possible to prevent malnutrition and its consequences, such as treatment delays, interruptions, and poor treatment outcomes. The current prospective study design demonstrated that the PNI and PLR combination was a robust prognostic indicator of one year mortality for LAHNC patients undergoing CCRT. These parameters are relatively simple, inexpensive, and easily accessible in clinical practice and are correlated with muscle loss measured by DXA.

## Figures and Tables

**Table 1:** Pretreatment baseline characteristics of LAHNC patients undergoing CCRT.

Variables	Total
	Numbers (%) or Mean ± SD
Included patient No.	113 (100)
Clinicopathological data	
Age (years)	53.6 ± 8.3
Gender (male : female)	110 (97.3) : 3 (2.7)
Smoking (no : yes)	8 (7.1) : 105 (92.9)
Alcohol (no : yes)	28 (24.8) : 85 (75.2)
Betel nut (no : yes)	42 (37.2) : 71 (62.8)
ECOG performance status (0 : 1 : 2)	9 (8.0) : 98 (86.7) : 6 (5.3)
HN-CCI (0 : 1 : 2)	72 (63.7) : 33 (29.2) : 8 (7.1)
Location (oral cavity : oropharynx : hypopharynx : larynx : unknown primary)	56 (49.6) : 25 (22.1) : 20 (17.7) : 10 (8.8) : 2 (1.8)
Histologic grade (well : moderate : poor : unknown)	7 (6.2) : 74 (65.5) : 21 (18.6) : 11 (9.7)
Tumor (T0 : T1 : T2 : T3 : T4)	2 (1.8) : 4 (3.5) : 20 (17.7) : 17 (15.0) : 70 (62.0)
Lymph node (N0 : N1 : N2 : N3)	20 (17.7) : 21 (18.6) : 64 (56.6) : 8 (7.1)
Stage, AJCC 7th edition (III : IVA : IVB)	12 (10.6) : 79 (69.9) : 22 (19.5)
Neoadjuvant chemotherapy (no : yes)	81 (71.7) : 32 (28.3)
Curative surgery (no : yes)	61 (54.0) : 52 (46.0)
Cisplatin dose (mg/m^2^)	216 ± 62
Radiation	
dose (Gy)	65.3 ± 10.9
days to finish	49.4 ± 11.2
Laboratory data	
Hemoglobin (g/dL)	11.9 ± 1.7
WBC count (× 10^9^/L)	7.4 ± 2.6
Platelet count (× 10^9^/L)	305.3 ± 130.0
Total lymphocyte count (× 10^9^/L)	1.7 ± 0.6
Albumin (g/dL)	3.9 ± 0.4
Nutrition Index	
BMI (kg/m^2^)	22.9 ± 4.3
BWL (< 10% : ≥ 10%)	84 (74.3) : 29 (25.7)
PNI	47.9 ± 5.9
NRI (no : mild : moderate : severe)	68 (60.2) : 11 (9.7) : 27 (23.9) : 7 (6.2)
PG-SGA (well (0–3) : moderate (4–8) : severe (≥ 9))	88 (77.9) : 24 (21.2) : 1 (0.9)
Inflammatory biomarkers	
NLR	3.4 ± 2.9
PLR	212.3 ± 149.5
LMR	3.6 ± 2.4
DXA-derived LBMI (kg/m^2^)	15.9 ± 2.0
1-year all-cause mortality rate	30.9%

Abbreviations: LAHNC, locally advanced head and neck cancer; CCRT, concurrent chemoradiotherapy; SD, standard deviation; ECOG, Eastern Cooperative Oncology Group; HN-CCI, Head and Neck Charlson Comorbidity Index; AJCC, American Joint Committee on Cancer; WBC, white blood cell; BMI, body mass index; BWL, body weight loss; PNI, prognostic nutritional index; NRI, nutritional risk index; PG-SGA, patient-generated subjective global assessment; NLR, neutrophil-to-lymphocyte ratio; PLR, platelet-to-lymphocyte ratio; LMR, lymphocyte-to-monocyte ratio; DXA, dual energy X-ray absorptiometry; LBMI, lean body mass index.

**Table 2:** Univariate and multivariate analyses of factors associated with 1 year mortality of LAHNC patients undergoing CCRT.

Variables	Total			
	Univariate		Multivariate	
	HR (95% CI)	*p*	HR (95% CI)	*p*
Age (< 65 y vs. ≥ 65 y)	1.246 (0.381–4.069)	0.716		
Gender (male vs. female)	1.094 (0.405–2.958)	0.859		
Smoking (no vs. yes)	1.255 (0.301–5.231)	0.755		
Alcohol (no vs. yes)	1.754 (0.728–4.225)	0.210		
Betel nut (no vs. yes)	1.878 (0.879–4.009)	0.104		
ECOG PS (< 2 vs. ≥2)	3.208 (1.123–9.163)	**0.029**		
HN-CCI (< 1 vs. ≥ 1)	0.564 (0.264–1.204)	0.139		
Location (oral cavity vs. non-oral cavity cancer)	0.377 (0.184–0.771)	**0.008**		
Histologic grade (well vs. moderate/poor)	0.481 (0.169–1.366)	0.169		
Tumor (T0–T2 vs. T3–T4)	2.576 (0.909–7.300)	0.075		
Lymph node (N0–N1 vs. N2–N3)	1.480 (0.711–3.081)	0.295		
Stage (III vs. IV)	1.315 (0.403–4.295)	0.650		
Neoadjuvant chemotherapy (no vs. yes)	0.950 (0.456–1.978)	0.891		
Curative surgery (no vs. yes)	2.295 (1.155–4.562)	**0.018**		
Cisplatin dose (< 160 vs. ≥ 160 mg/m^2^)	0.893 (0.347–2.303)	0.815		
Radiation Dose (< 66 vs. ≥ 66 Gy) Days (< 50 vs. ≥ 50 d)	0.806 (0.413–1.575) 1.320 (0.680–2.561)	0.528 0.412		
Hemoglobin (< 10 vs. ≥ 10 g/dL)	0.423 (0.192–0.931)	**0.033**		
WBC count (< 11.0 vs. ≥ 11.0 × 10^9^/L)	1.472 (0.611–3.548)	0.389		
Platelet count (< 400 vs. ≥ 400 × 10^9^/L)	1.975 (0.981–3.976)	0.057		
TLC (< 1.5 vs. ≥ 1.5 × 10^9^/L)	0.297 (0.149–0.590)	**0.001**		
Albumin (< 3.5 vs. ≥ 3.5 g/dL)	0.294 (0.141–0.617)	**0.001**		
BMI (< 18.5 vs. ≥ 18.5 kg/m^2^)	0.296 (0.144–0.608)	**0.001**		
BWL (< 10% vs. ≥ 10%)	1.640 (0.815–3.298)	0.165		
PNI (< 46.8 vs. ≥ 46.8) *	0.199 (0.097–0.409)	**<0.001**	0.276 (0.131–0.582)	**0.001** **^#^**
NRI (< 97.5 vs. ≥ 97.5)	0.387 (0.199–0.753)	**0.005**		
PG-SGA (well vs. moderate/severe)	1.791 (0.876–3.661)	0.110		
NLR (< 3.5 vs. ≥ 3.5) *	4.078 (2.089–7.962)	**<0.001**		
PLR (< 191 vs. ≥ 191) *	4.585 (2.238–9.393)	**<0.001**	3.205 (1.520–6.757)	**0.002** **^#^**
LMR (< 2.1 vs. ≥ 2.1) *	0.384 (0.193–0.764)	**0.006**		
LBMI (< 14.4 vs. ≥ 14.4 kg/m^2^) ^†^	0.496 (0.249–0.986)	**0.045**		

* ROC curves were used to determine the optimal cutoff values for the PNI, NLR, PLR, and LMR. ^†^ The lowest quartile was used as the cutoff value for the LBMI. Bold represents a significant *p*-value. ^#^ Adjusted for ECOG PS, location, surgery, hemoglobin, TLC, albumin, BMI, NRI, NLR, LMR, and LBMI. Abbreviations: LAHNC, locally advanced head and neck cancer; CCRT, concurrent chemoradiotherapy; ECOG PS, Eastern Cooperative Oncology Group performance status; HN-CCI, Head and Neck Charlson Comorbidity Index; WBC, white blood cell; TLC, total lymphocyte count; BMI, body mass index; BWL, body weight loss; PNI, prognostic nutritional index; NRI, nutritional risk index; PG-SGA, patient-generated subjective global assessment; NLR, neutrophil-to-lymphocyte ratio; PLR, platelet-to-lymphocyte ratio; LMR, lymphocyte-to-monocyte ratio; LBMI, lean body mass index.

## Supplementary Materials

The following are available online at https://www.mdpi.com/2072-6643/12/3/836/s1: Figure S1: Kaplan–Meier survival curve showing survival differences of four risk combination groups of PNI and PLR, Figure S2: The linear relationships between the PNI (A) and PLR (B) and LBMI, Table S1: Pretreatment malnutrition rates of 113 LAHNC patients undergoing CCRT.
