# Supplementary material for: Determining Malnutrition Assessment Criteria to Predict One-Year Mortality for Locally Advanced Head and Neck Cancer Patients Undergoing Concurrent Chemoradiotherapy
Source: Nutrients. 2020 Mar 20;12(3):836. doi: 10.3390/nu12030836 (PMC7146124; doi:10.3390/nu12030836)
Supplement: Supplementary file 1 [file nutrients-12-00836-s001.pdf]

Table S1. Pretreatment malnutrition rates of 113 LAHNC patients undergoing CCRT.

Variables	Numbers (%)
BMI <18.5 kg/m^2	17 (15.0)
BWL ≥10%	29 (25.7)
TLC <1.5 x 10^9/L	45 (39.8)
Albumin <3.5 g/dL	15 (13.3)
PNI <46.8	42 (37.2)
NRI <97.5	34 (30.1)
PG-SGA<br>(moderate or severely malnourished)	25 (22.1)
NLR ≥3.5	32 (28.3)
PLR ≥191	44 (38.9)
LMR <2.1	24 (21.2)

Abbreviations: LAHNC, locally advanced head and neck cancer; CCRT, concurrent chemoradiotherapy; BMI, body mass index; BWL, body weight loss; TLC, total lymphocyte count; PNI, prognostic nutritional index; NRI, nutritional risk index; PG-SGA, patient-generated subjective global assessment; NLR, neutrophil-to-lymphocyte ratio; PLR, platelet-to-lymphocyte ratio; LMR, lymphocyte-to-monocyte ratio.

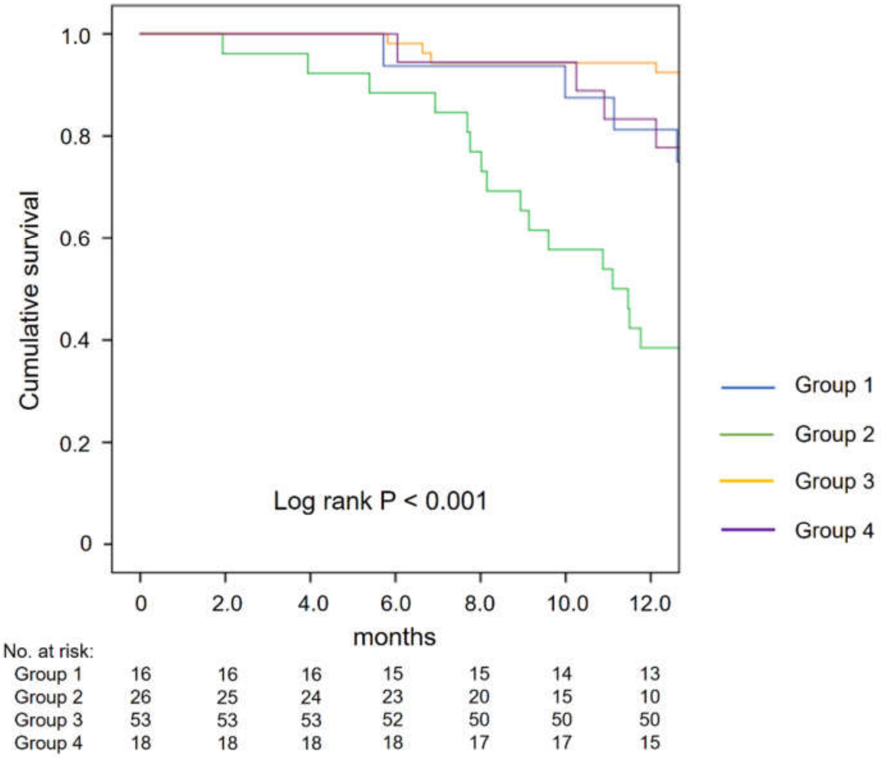

Figure S1. Kaplan-Meier survival curve showing survival differences of 4 risk combination groups of PNI and PLR. Group 1, low PNI/low PLR (n=16); Group 2, low PNI/high PLR (n=26); Group 3, high PNI/low PLR (n=53); Group 4, high PNI/high PLR (n=18).

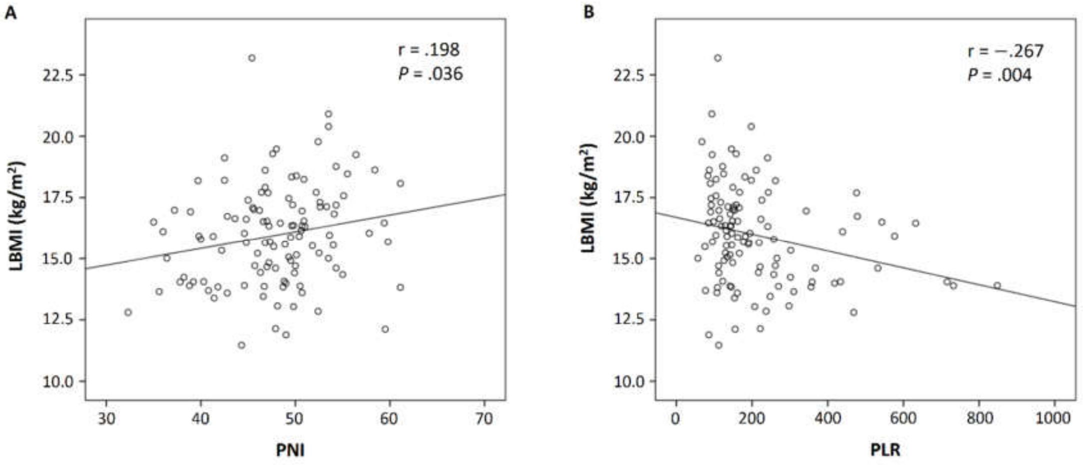

Figure S2. The linear relationships between the PNI (A) and PLR (B) and the LBMI. LBMI, lean body mass index; PNI, prognostic nutritional index; PLR, platelet-to-lymphocyte ratio.
